# *Bartonella bacilliformis*: A Systematic Review of the Literature to Guide the Research Agenda for Elimination

**DOI:** 10.1371/journal.pntd.0001819

**Published:** 2012-10-25

**Authors:** Nuria Sanchez Clemente, Cesar A. Ugarte-Gil, Nelson Solórzano, Ciro Maguiña, Paul Pachas, David Blazes, Robin Bailey, David Mabey, David Moore

**Affiliations:** 1 London School of Hygiene and Tropical Medicine, London, United Kingdom; 2 Instituto de Medicina Tropical Alexander van Humboldt, Universidad Peruana Cayetano Heredia, Lima, Peru; 3 Hospital San Juan de Dios, Caraz, Peru; 4 DoD Global Emerging Infections System, Armed Forces Health Surveillance Center, Silver Spring, Maryland, United States of America; University of Texas Medical Branch, United States of America

## Abstract

**Background:**

Carrion's disease affects small Andean communities in Peru, Colombia and Ecuador and is characterized by two distinct disease manifestations: an abrupt acute bacteraemic illness (Oroya fever) and an indolent cutaneous eruptive condition (verruga Peruana). Case fatality rates of untreated acute disease can exceed 80% during outbreaks. Despite being an ancient disease that has affected populations since pre-Inca times, research in this area has been limited and diagnostic and treatment guidelines are based on very low evidence reports. The apparently limited geographical distribution and ecology of *Bartonella bacilliformis* may present an opportunity for disease elimination if a clear understanding of the epidemiology and optimal case and outbreak management can be gained.

**Methods:**

All available databases were searched for English and Spanish language articles on Carrion's disease. In addition, experts in the field were consulted for recent un-published work and conference papers. The highest level evidence studies in the fields of diagnostics, treatment, vector control and epidemiology were critically reviewed and allocated a level of evidence, using the Oxford Centre for Evidence-Based Medicine (CEBM) guidelines.

**Results:**

A total of 44 studies were considered to be of sufficient quality to be included in the analysis. The majority of these were level 4 or 5 (low quality) evidence and based on small sample sizes. Few studies had been carried out in endemic areas.

**Conclusions:**

Current approaches to the diagnosis and management of Carrion's disease are based on small retrospective or observational studies and expert opinion. Few studies take a public health perspective or examine vector control and prevention. High quality studies performed in endemic areas are required to define optimal diagnostic and treatment strategies.

## Author Summary

Carrion's disease is one of the truly neglected tropical diseases. It affects children predominantly in small Andean communities in Peru, Colombia and Ecuador. Case fatality rates of untreated acute disease can exceed 80% during outbreaks. Diagnostic and treatment guidelines are based on very low evidence reports and public health and prevention programs have been limited. This paper presents the first systematic review of Carrion's disease in Peru and encompasses a detailed analysis of all the highest level evidence regarding not only diagnosis and management but also vector control and prevention. In the review, the authors highlight the considerable knowledge gaps in this field and suggest a strategy for a renewed effort in its investigation. The authors hope that through this work we will be able to develop a better understanding of the epidemiology, natural history and optimal approaches to case and outbreak

## Introduction

*Bartonella bacilliformis* is a gram negative, facultative intracellular, aerobic coccobacillus which is a member of the alpha-proteobacteria group along with *Rickettsia* and *Brucella* [1]. It is responsible for a spectrum of disease which, despite its limited distribution, has been given a multitude of names including bartonellosis, Carrion's disease, Oroya fever and verruga peruana.

**Figure 1:**
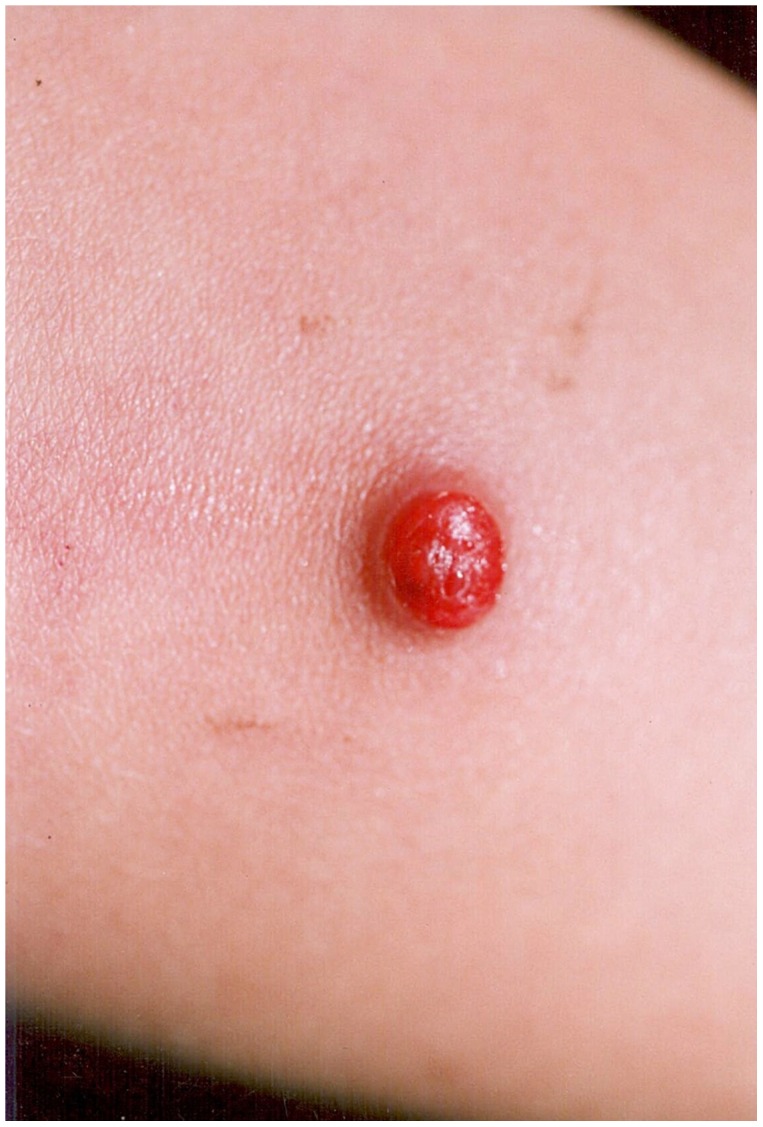
Miliary lesion. Courtesy of C. Maguiña.

**Figure 2:**
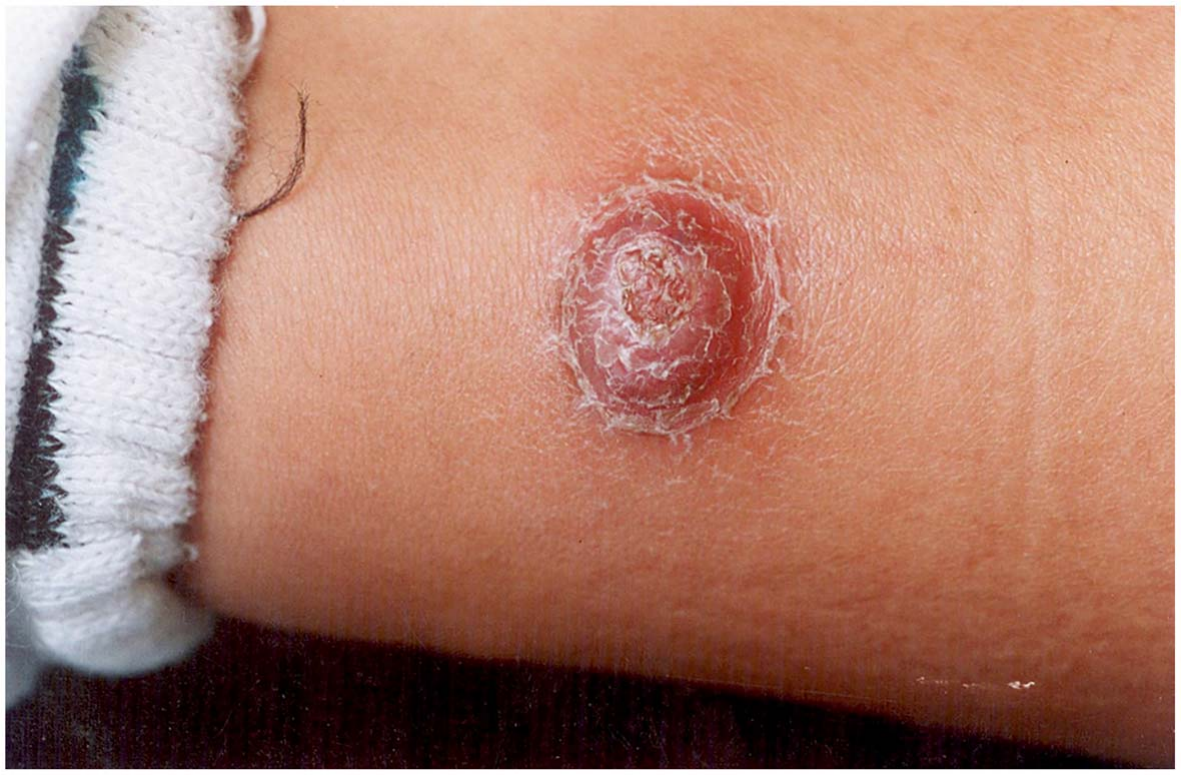
Mular lesion. Courtesy of C. Maguiña.

**Figure 3:**
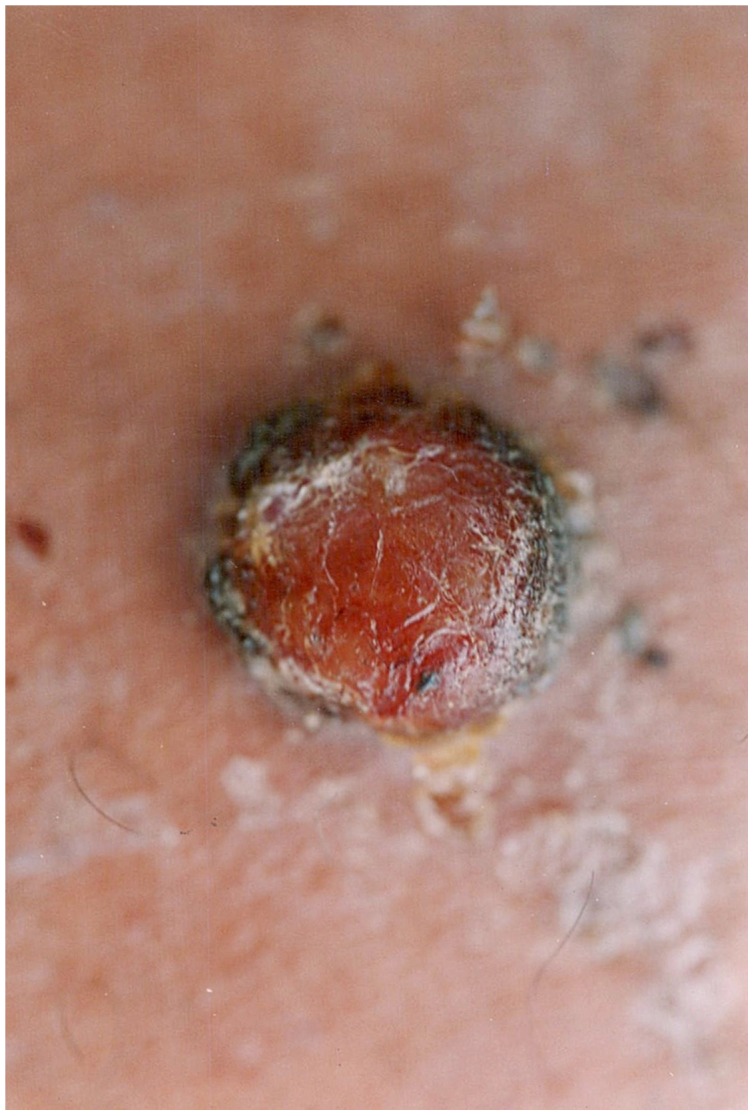
Mular lesion. Courtesy of C. Maguiña.

The organism causes two distinct clinical syndromes. The initial acute phase is characterised by fever and haemolytic anaemia and has a reported mortality of 44% to 88% in untreated individuals [2]. The subsequent phase, which may occur weeks to months after the acute illness (and there may or may not be a history of antecedent illness), is characterised by the eruption of crops of miliary (figure 1), mular (figures 2 and 3) or nodular skin lesions, or verrugas (“warts”), containing sero-sanguinous fluid which exudes on contact.

Complications are not uncommon in the acute form and include super-infections, most commonly with *Salmonella* species but also with *Toxoplasma*, *Histoplasma* and others [3], [4]. Haematological, gastrointestinal [5], cardiovascular [6] and neurological [7] complications also occur and in pregnancy, infection can lead to miscarriage, premature labour and maternal death. Young children are the most affected in endemic communities, partly because of a predominantly younger population but also due to the presumed protective immunity that develops with repeated infection [8].

**Figure 4:**
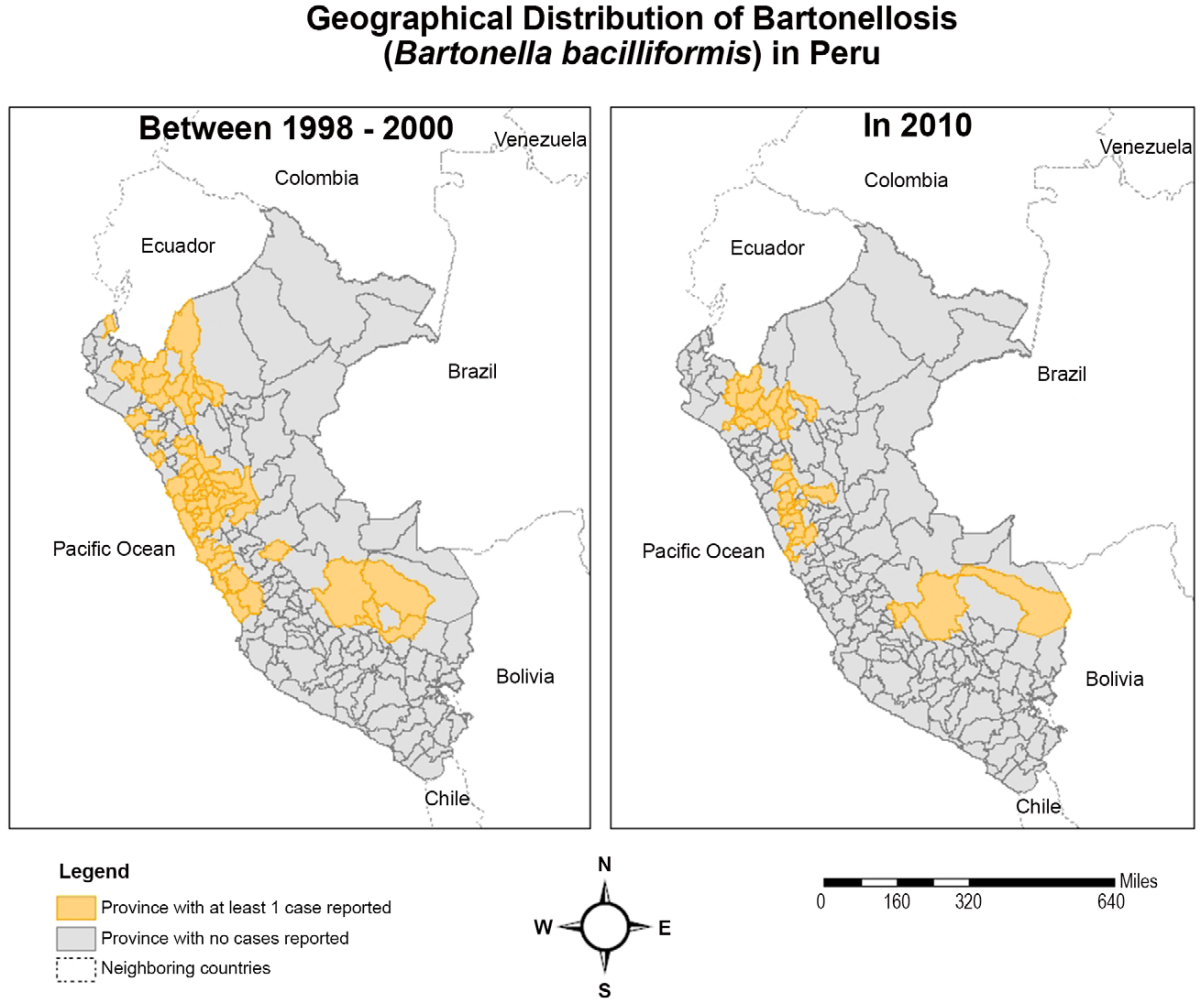
Geographical distribution of bartonellosis in Peru. Courtesy of Ricardo Castillo (JHSPH).

The disease is restricted to the Andean cordillera in Peru (figure 4), Ecuador, and Colombia with unconfirmed reports of cases in Thailand in the 1960s [9] and sporadic cases in Bolivia, Chile and possibly Guatemala [10]. Classically, endemic areas are said to be confined to inter-Andean valleys positioned at right-angles to the prevailing wind [9] and at altitudes between 500 to 3200 m above sea level [11]. This focality is mainly due to the characteristics of its putative principal vector, *Lutzomyia verrucarum* which has a weak, hopping flight and is intolerant of extreme temperatures [9]. The vector has a crepuscular, endophilic feeding habit and households are heterogeneously affected, with 18% of households accounting for 70% of cases in one series [12]. It is thought that El Niño events, which cause a warming in sea temperature every 5–7 years, favourably affect vectors due to a change in climatic conditions [13].

The history of the disease retains a degree of mystery as it has not been fully established when the illness was first recognised. Paleodiagnosticians still debate whether it was Carrion's disease that was responsible for a devastating epidemic in Huayna Capac's Incan empire which killed 200,000 of his people shortly before the Spanish invasion and there remains debate about whether it was this disease which killed almost half of Pizarro's men in Coaque during the Spanish colonization of Ecuador in 1532 [9].

The eponym Carrion's disease recognises the contribution of Daniel Alcides Carrion, a Peruvian medical student who in 1885 asked a fellow student to inoculate him with blood from a warty cutaneous lesion from a diseased patient, in order to test his hypothesis that the two clinical entities, which were considered at the time to be different illnesses, were actually manifestations of the same disease. His hypothesis was proven to be tragically correct as he developed, and soon after succumbed to, the acute febrile form of the illness hitherto known as Carrion's disease, becoming a martyr of Peruvian medicine [14].

This rich history is evident when visiting endemic areas as a number of Quechua words exist for describing the disease and traditional healers, or curanderos, have developed a range of remedies for treating the disease.

Due to its focal geographical nature and the fact that it affects small, isolated, rural communities, Carrion's disease has been truly neglected. Diagnostic and treatment guidelines [15], [16] are supported only by very low evidence studies and expert opinion. However there are early suggestions of an expanding ecological niche and the window of opportunity for disease elimination may be starting to close; if definitive evidence-driven control efforts are to succeed it will be important to quickly identify and address important knowledge gaps.

## Materials and Methods

### Search methods

The following databases were used to search for articles in both English and Spanish language from June to August 2010. EMBASE, Global Health on OVID, HISA, LEYES, LILACS, MEDLINE on OVID, PubMed, REPIDISCA, BASE and ADOLEC.

References of key papers were examined for supplementary studies. In addition, local experts in the field were contacted to supply any further abstracts, unpublished data and conference proceedings.

Only articles in English and Spanish were included in the systematic review. No limits were placed on year of publication.

The following search terms were used:

Bartonel?osis

Bartonella AND bacilliformis

Verruga AND peru??ana

Carrion* AND disease

Oroya AND Fever

The articles obtained were transferred to EndNote reference manager and checked for duplicates. The final *Bartonella bacilliformis* library contained 480 articles. This list was further scrutinized to select those of interest for the systematic review.

**Figure 5:**
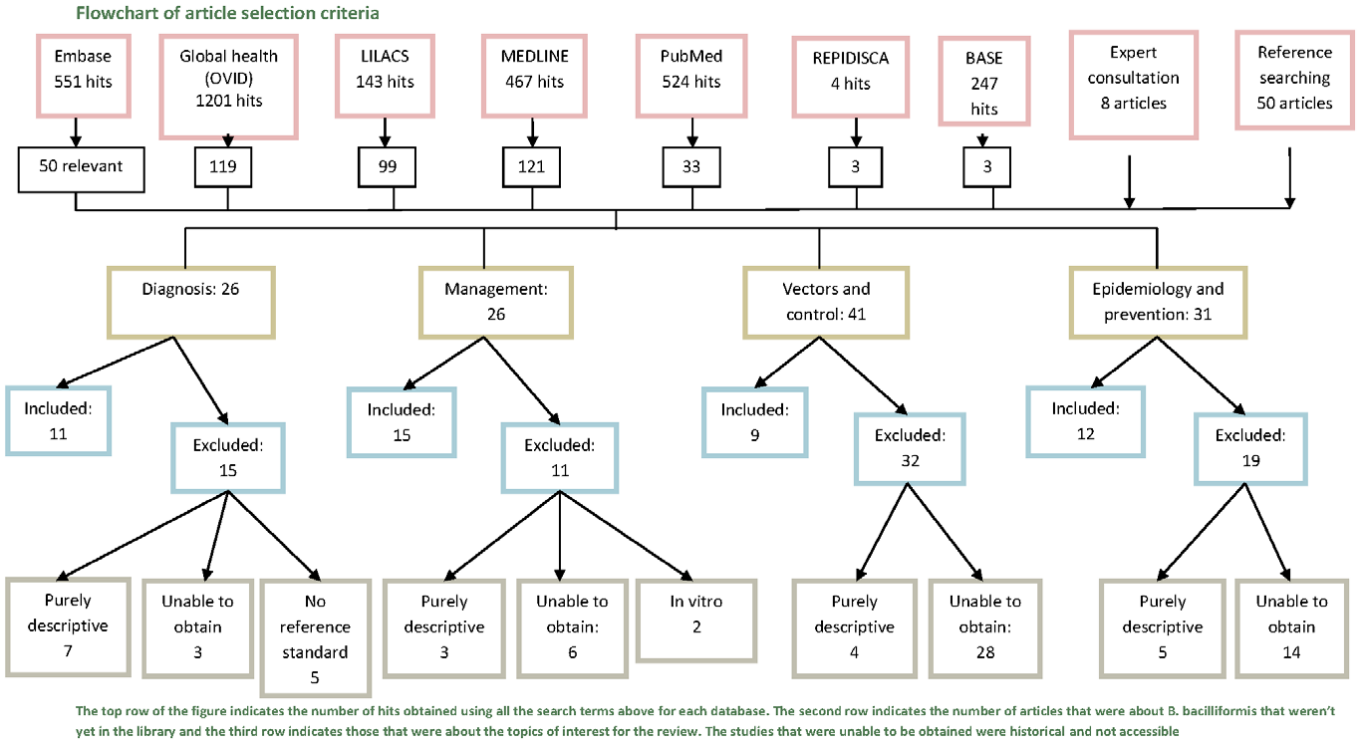
Flowchart of article selection criteria.

Further details on the search and selection method are provided in Figure 5.

### Inclusion criteria

The preliminary search was carried out to compile a comprehensive *Bartonella bacilliformis* library. Due to the fact that high-level evidence on the subject is limited, all types of trials and articles were considered for inclusion.

The Endnote library was consulted for trials looking at diagnosis, management, prevention, epidemiology and control of *B. bacilliformis*. This search obtained 26 articles relating to diagnosis, 26 on management, 41 on vectors and control and 31 about epidemiology and prevention.

Articles that were purely descriptive without any analysis, were excluded from the review. In vitro studies looking at antibiotic effectiveness were excluded unless this was the only data available regarding that particular antibiotic. Diagnostic studies that did not use a reference standard were also excluded. Some historical articles could not be sourced. Articles that contained duplicated data were excluded.

A total of 47 studies were considered to be of sufficient quality to be included in the review; 11 on diagnosis, 15 on management, 9 on vectors and control and 12 on epidemiology and control. A flow diagram of the selection process compiled using PRISMA (Preferred Reporting Items for Systematic Reviews and Meta-Analyses) guidelines can be seen as part of the supporting documents (Figure S1).

All articles were rated according to level of evidence using the Oxford Centre for Evidence-based Medicine's Levels of Evidence, March 2009 [17]. Please see Figure S3 for details. The full EndNote library is freely available upon request.

## Results

The principal characteristics and salient outcomes of the studies are summarized in tables 1, 2, 3, and 4 according to subject.

**Table 1:** Summary of studies on diagnosis and their level of evidence.

Reference	Diagnostic test	Reference standard	N	Study design and location	Type of disease	Outcome	Level of evidence
**Chamberlin, 2000 ** [**19**]	IFA	Blood culture or smear	134	Case-control, Caraz	Unspecified	Sensitivity: 85% (28/33) Specificity: 92% (93/101) Positive predictive value: 89%	3b
**Knobloch, 1985 ** [**20**]	IFA	ELISA	102	Case series, Cajamarca	Unspecified	Sensitivity: 51% (52/102)	4
**Padilla, 2003 ** [**21**]	PCR	Blood smear	15	Case-control, Ancash, Cuzco, Lima	Acute	Sensitivity: 100% (10/10) Specificity: 100% (5/5)	4
**Padilla, 2006 ** [**22**]	ELISA	Blood culture, smear or PCR	77	Case-control, Lima	24 acute, 3 chronic	Sensitivity: IgM-85% (23/27), IgG-70% (20/27) Specificity: 100% (40/40)	3b
**Maguiña, 2001 ** [**23**]	ELISA	Blood culture	9	Case series, Lima	Acute	Sensitivity: 89% (8/9)	4
**Mallqui, 2000 ** [**24**]	Sonicated immunoblot	Blood smear or biopsy	122	Case-control, Lima	10 Acute, 32 chronic	Sensitivity: Acute: 30–70% (3/10–7/10), Chronic: 94% (30/32), Specificity: 100% (80/80)	3b
**Ellis, 1999 ** [**25**]	Thin blood smear	PCR	146	Case-control, Urubamba region	Acute	Sensitivity: 36% (4/11) Specificity: 96% (125/130)	3b
**Pachas, 2004 ** [**26**]	Thin blood smear	PCR or blood culture	778	Case series, Caraz	624 acute, 154 chronic	Sensitivity: 24%	3b
**Maguiña, 2002 ** [**27**]	Western blot	‘Clinical expert’	11	Case series, Caraz	Chronic	Sensitivity: 100% (11/11)	4
**Maguiña, 2001 ** [**23**]	Western blot	Blood culture	9	Case series, Lima	Acute	Sensitivity: 100% (9/9)	4

**Table 2:** Summary of the studies on management and their level of evidence.

Reference	Treatment	N	Study design and location	Type of disease	Outcome	Level of evidence
**Minnick, 2003 ** [**28**] ** Angelakis, 2008 ** [**30**]	Ciprofloxacin	N/A	In vitro	N/A	Constitutive mutations in *B. Bacilliformis* causing resistance to cipro identified	5
**Minnick, 2003 ** [**28**] ** Sobraques, 1999 ** [**29**]	Ciprofloxacin	N/A	In vitro	N/A	MIC = 0.25	5
**Biswas, 2007 ** [**31**] ** Del Valle, 2010 ** [**32**]	Ciprofloxacin	N/A	In vitro	N/A	*B. bacilliformis* quickly becomes resistant to cipro	5
**Urteaga, 1955 ** [**34**]	Chloramphenicol, IV	19	Case series, Lima	Acute	79% made a ‘rapid recovery’	4
**Arroyo, 2004 ** [**35**]	Chloramphenicol	215	Observational study, Caraz	Acute	89% achieved clinical cure	4
**Maguiña, 2001 ** [**23**]	Chloramphenicol	65	Case series, Lima	Acute	95.4% responded well	4
**Rolain, 2004 ** [**33**]	Chloramphenicol	52	Minireview	Acute	Good evidence for use from one or more well-designed clinical trials	3a
**Hodgson, 1947 ** [**36**]	Large blood transfusions	2	Case series, Lima	Acute	Both survived	4
**Arroyo, 2004 ** [**35**]	Rifampicin	260	Observational study, Caraz	Chronic	93.1% achieved clinical cure	4
**Maguiña, 2001 ** [**23**]	Rifampicin	46	Case series, Lima	Chronic	80% responded well	4
**Rolain, 2004 ** [**33**]	Rifampicin	46	Minireview	Chronic	Good evidence for use from one or more well-designed clinical trials	3a
**Rolain, 2004 ** [**33**]	Streptomycin	9	Minireview	Chronic	Good evidence for use from one or more well-designed clinical trials	3a
**Gonzalez, 2003 unpublished ** [**37**]	Rifampicin vs. azithromycin	127	Case-control, Caraz	Chronic	Time to negative cultures for both was 3–4 weeks	5
**Gutierrez, 1998 ** [**38**]	Sultamicilline and deflazacort	1	Case report	Chronic	Successfully treated	5

**Table 3:** Summary of the studies on vectors and control and their level of evidence.

Reference	Species/Control method	Outcomes	Study Location	Level of evidence
**Montoya, 1998 ** [**39**]	*L. peruensis* and *L. pescei*	Found in large and small quantities respectively	Cusco	4
**Ellis, 1999 ** [**25**]	*L. peruensis*	*B. bacilliformis* detected in 2% (2 of 104) of specimens by PCR	Urubamba/Cusco region	3b
**Ponce, 2002 ** [**42**]	*L.. verrucarum*	Unable to conclude whether vertical transmission occurs as sample too small	Caraz	3b
**Ponce, 2002 ** [**42**]	*L. verrucarum*	There is a significant difference between lifespan and degree of oviposition between sandflies infected with low bacteraemic and high bacteraemic blood. (p<0.001)	Caraz	3b
**Caceres, 1997 ** [**40**]	*Lu. robusta* and *Lu. maranonensis*	Found in intra-domiciliary areas	San Ignacio, Jaen and Utcubamba	4
**Tejada, 2003 ** [**41**]	*L. serrana*	93.6% of sandflies collected	Huanuco	4
**Hertig, 1948 ** [**43**]	DDT	Treatment of stone walls and house spraying reduced numbers of vectors	Rimac Valley	4
**Solorzano, 2009 unpublished ** [**44**]	Pyrethroids	Pyrethroids may have additional irritant and repellent effects which last longer than the insecticide	Choquechaca, Yuracoto, Caraz, Cullashpampa	5

**Table 4:** Summary of the studies on epidemiology and prevention and their level of evidence.

Reference	Topic	N	Main Outcomes	Study Location	Level of evidence
**Ricketts, 1947 ** [**45**]	Incubation period	13	19–100 days	Lima	4
**Howe, 1942 ** [**46**]	Prophylactic immunisation	32	55% of vaccinated developed positive cultures vs. 90% of unvaccinated	Ancash	3b
**Chamberlin, 2002 ** [**13**]	Asymptomatic bacteraemia	555	Point prevalence in January 1997: 0.5%	Huaylas province	1b
**Chamberlin, 2002 ** [**13**]	Prevalence of past infection	555	45% of the population	Huaylas province	1b
**Chamberlin, 2002 ** [**13**]	Incidence of infection	555	12.7 per 100 person years	Huaylas province	1b
**Herrer, 1953 ** [**47**]	Asymptomatic bacteraemia	14	Unable to culture B. bacilliformis with regularity from asymptomatic cases	San Juan	4
**Chinga-Alayo, 2004 ** [**14**]	The El Niño phenomenon	N/A	Significant difference in monthly bartonellosis incidence between el Niño and non-el Niño periods	Cusco and Ancash	3b
**Huarcaya Castilla, 2004 ** [**48**]	The El Niño phenomenon	N/A	There was a 4-fold increase in monthly cases in El Niño periods	Ancash	3b
**Cooper, 1997 ** [**50**]	Rats as a reservoirs for bartonellosis	48	Peri-domicilliary dead rodents were found more in case than control houses (p<0.05)	Zamora Chinchipe province, Ecuador	4
**Cooper, 1996 ** [**51**]	Chickens as a reservoirs	8	Sick or dead chickens were found in increased frequency in case compared to control households (p<0.05)	Zamora Chinchipe province, Ecuador	4
**Birtles, 1999 ** [**49**]	Domestic and peri-domestic animals as reservoirs	50	None of the 41 domestic animals were infected. 5 of 9 other animals had Bartonella-like organisms in blood	Huayllacallan valley, Ancash	4
**Herrer, 1953 ** [**48**]	Euphorb plants as reservoirs	N/A	Unable to recover *B. bacilliformis* from the plants, infect seedlings or grow the microorganism in the presence of latex dilutions.	Ancash	5

### Diagnosis (table 1)

Evaluations of diagnostics generally provide low quality evidence and are plagued by ill-defined reference standards, inappropriate control groups, frequent failure to disaggregate diagnostic performance in acute and chronic forms of disease and lack of application of currently accepted STARD guidelines [18].

#### Indirect fluorescence antibody test (IFA)

Two studies examined the diagnostic efficacy of IFA. The first, by Chamberlin et al [19], tested 33 cases (blood culture or smear positive) and 101 controls and found a sensitivity of 85% and a specificity of 92%. Four patients infected with other pathogens were tested for cross-reactivity (two with syphilis, two with cat-scratch disease, caused by *Bartonella henselae*) and one of each tested positive.

The second, by Knobloch et al [20], looked at 187 sera obtained from patients resident in an endemic area of the Northern district of Cajamarca and showed a much lower sensitivity of 45% when compared to ELISA as a reference standard.

#### PCR

The only study found looking at PCR which used a reference standard was by Padilla et al. [21] All 10 smear-positive blood samples tested positive for B. bacilliformis and 5 additional malaria positive samples were correctly identified as being negative for Bartonella.

#### ELISA

The larger of two ELISA evaluation studies [22] compared 27 lab-confirmed cases (by blood culture, smear or PCR) to 40 healthy controls who were not from, and had never visited endemic areas; and 10 sera of patients known to have different infections. Sensitivity using IgM was superior to IgG ELISA (85% vs 70%); specificity was 100% for both. One patient with Salmonella infection tested positive but none of the other pathogens cross-reacted with the test.

Eight of nine smear-positive patients tested by Maguiña [23] between 1969 and 1992 had positive ELISA.

#### Sonicated immunoblot

Mallqui et al. [24] tested this technique on 42 confirmed Carrion's disease patients (blood smear or biopsy positive). Two methods of antigen preparation were tested; sonicated and glycine. For chronic disease the sensitivity was 94% using both, whereas for acute cases, sensitivity was 70% for sonicated and 30% for glycine. The specificity was quoted as 100% for both when sera of healthy volunteers were tested with immunoblot compared to blood smear. However, when testing sera known to be positive for brucellosis, *C. psittaci* and *Coxiella burnettii*, 34%, 5%, and 29% of the sera respectively cross-reacted and gave a false positive result.

#### Thin blood smear

Despite being the oldest and most widely employed method of diagnosis of *B. bacilliformis*, only two published studies have looked at the sensitivity and specificity of this test. Ellis et al. [25] found that out of 11 PCR positive acute cases tested during the 1998 outbreak in the Urubamba region, only 4 were thin smear positive, giving a sensitivity of 36%, and a positive predictive value of 44%. A specificity of 96% was obtained as 125 individuals were thin smear negative out of 130 PCR negative individuals.

Pachas et al. [26] found that of 352 confirmed acute and chronic cases in Caraz (Ancash, Peru), 24% were thin smear positive.

#### Western blot

Two studies by Maguiña et al. have looked at Western blot compared to reference standard. One [27] used a ‘clinical expert’ to make a clinical diagnosis in 11 chronic patients and the other [23] tested 9 culture-positive patients with acute disease. All of these tested positive for *B. bacilliformis* with Western blot.

### Management – acute disease (table 2)

There are no published controlled clinical trials of therapy for acute or chronic Carrion's Disease and interpretation of observational data is often complicated by lack of a standardized case definition, lack of an adequate comparator arm, weak outcome definitions and outcome ascertainment.

#### Ciprofloxacin

Studies of quinolone therapy have reached divergent conclusions.

Minnick [28] and Sobraques et al. [29] in separate studies published in 2008, found ciprofloxacin to have a minimum inhibitory concentration (MIC) of 0.25 but disagreed on whether this was high or low, with Sobraques [29] concluding that the organism was highly susceptible to ciprofloxacin and Minnick [28] discouraging its use, a suggestion purportedly later substantiated by the description of constitutive mutations in the quinolone-resistance-determining region of gyrase (gyrA) by Minnick [28] and Angelakis [30].

Biswas [31] and del Valle [32] found that *B. bacilliformis* can quickly become resistant to ciprofloxacin *in vitro* and therefore concluded that its use should be discouraged in the treatment of Carrion's Disease, whilst Rolain [33] concluded that there was ‘moderate evidence for the use of ciprofloxacin from opinions of respected authorities.’ This was based on unpublished reports.

#### Chloramphenicol

Three in vivo studies have been carried out looking at the effectiveness of chloramphenicol, the first of which was carried out by Urteaga [34] in the 1950s and involved 19 cases. Although the results reported that the majority, 79% (15), made a rapid recovery, the study lacked details of sampling methods and doses used.

Chloramphenicol was the most used antibiotic in the acute phase in a cohort of 518 patients studied by Arroyo in Caraz [35]. The treatment resulted in clinical cure in 89%. However, none of the patients received the recommended loading dose of 50 mg/kg and 66.1% required a higher maintenance dose of 25 mg/kg and indeed 39.1% required a prolonged treatment course greater than 14 days.

A similar study by Maguiña [23] carried out in Lima found that 95.4% of acute patients who had received chloramphenicol, either alone or with another antibiotic, responded well though disaggregated data for mono- and poly-microbial treatment was not presented.

Rolain et al. [33] classify chloramphenicol as having good evidence for use from one or more well-designed clinical trials, though this is based on 2 observational studies [10], [23] which lacked in depth analysis.

#### Large blood transfusions

One historical case-series by Hodgson [36] looked at the treatment of 2 acute cases with large blood transfusions. The patients survived but took 32 and 45 days to recover as well as 3.4 L and 8.15 L of blood respectively.

### Management – chronic disease (table 2)

#### Rifampicin and streptomycin

Arroyo [35], in his study of 518 cases, found that rifampicin was the most popular treatment for chronic disease leading to clinical cure in 93.1% of patients, though a large proportion (82%) required a prolonged treatment course of greater than 21 days.

Rolain [33] reported rifampicin as having ‘good evidence for use from one or more well-designed clinical trials’; this conclusion is based on one observational study carried out by Maguiña et al. [23] between 1969 and 1992 which found that 80% (37 of 46) of patients treated with rifampicin had a good response compared with 56% (5 of 9) who received streptomycin. From this data Rolain et al. maintained that the strength of evidence for streptomycin was the same as for rifampicin.

#### Rifampicin and azithromycin

In 2003, a group of researchers from Caraz carried out a study [37] (as yet unpublished) comparing azithromycin, the current 1^st^-line treatment, to rifampicin in 127 cases. The definition of cure used was reversion to negative blood cultures. Both antibiotics were equally efficacious and achieved a similar ‘cure time’ of 3 to 4 weeks.

#### Sultamicillin and deflazacort

A single case report [38] was published after the successful treatment of a 12-year old girl with chronic verruga with 21 days of treatment (25 mg/kg of sultamicillin, 0.7 mg/kg of deflazacort).

### Vectors and control (table 3)

Though it is widely held that *B. bacilliformis* is transmitted by the bite of an infected sandfly, evidence supporting this belief is remarkably lacking.

#### Vector species

A number of studies have attempted to implicate the *Lutzomyia* sandfly species in the transmission of Carrion's disease in different areas; most of them have involved CDC light trap collections during outbreaks. However, since few of them have identified *B. Bacilliformis* in the insect, the majority only present circumstantial evidence that these sandfly species could be the responsible vectors.

*L. peruensis* and, in smaller quantities, *L. pescei* were the only species found in a study carried out during an outbreak in Cusco [39]. Similar findings were reported by Ellis [25] who found only *L. peruensis* during an outbreak in the Cuzco region. In this second study, *B. bacilliformis* was identified using PCR in 2% of specimens collected (n = 2).

A further study carried out by Caceres [40] found a great abundance of *Lu. robusta* and *Lu. maranonensis* in intradomiciliary areas in the provinces of Jaen, San Ignacio and Utcubamba, however their possible role as vectors was not confirmed by PCR.

A different species, *L. serrana* was found to make up 93.6% of vectors collected in indoor CDC light traps and outdoor Shannon traps during an outbreak in Huamalíes, Huánuco in the high jungle area of Peru. However, once again carriage of *B. bacilliformis* by the sandflies could not be confirmed [41].

#### Vector characteristics

One study looking at vectorial characteristics of *L. verrucarum* tried to ascertain whether vertical transmission of *B. bacilliformis* occurred between adults and their offspring [42]. The investigators were unable to make firm conclusions regarding this, but they did find that the lifespan and degree of oviposition in sandflies infected with low bacteraemic blood(3%) was much longer and higher respectively compared with those fed on high bacteraemic (80%) blood (p<0.001).

#### Control

Studies looking at the control of *Lutzomyia* are scarce and none relate *Lutzomyia* control to Carrion's disease incidence. The first study was carried out in 1945 by Hertig and Fairchild [43] in the Rimac Valley, about 40 miles from Lima, where *L. verrucarum* and *L. peruensis* are abundant. Treatment of stone walls with DDT produced a marked reduction of sandflies, not quantified in the report, and treatment of stone walls combined with house spraying reduced sandflies to an ‘extremely low level.’

The only other study found was a report of the control programme in Caraz which took place between 2004 and 2008. The initiative involved indoor house spraying with residual insecticides [44]. The main conclusions drawn from this unpublished report were that intra-domiciliary sandfly populations remained remarkably low for over a year after spraying. This was unexpected as these insecticides were marketed to have an efficacy of 6 months. It is proposed that pyrethroids have additional irritant and repellent effects on the vectors that last longer than their known insecticide properties.

### Epidemiology and prevention (table 4)

#### Incubation period

In 1947 Ricketts [45] studied a group of patients who had recently visited endemic areas and proposed that their incubation periods, as determined by culture positivity, ranged from 20 to 100 days.

#### Immunisation

In 1943 Howe and his team [46] carried out a case control study using military staff posted out to verruga zones to test a crude vaccine made of 4 inactivated strains of *B. bacilliformis*. Out of 22 vaccinated men, 55% had developed positive blood cultures by the end of their duty period. This was in contrast to the control group, 90% (9 of 10) of which developed positive cultures over a similar period of time.

#### Asymptomatic infection

In 1997, Chamberlin [12] and her team in Caraz carried out a prospective cohort study which firstly used PCR to detect the number of asymptomatic bacteraemics in an endemic population; 0.5% of 555 were PCR positive. The study population was also tested for past infection using IFA and 45% had positive antibodies. The investigators then followed an abacteraemic cohort for 25 months monitoring them for clinical features of Carrion's disease. 127 cases were diagnosed in 25 months (12.7 per 100 person years) and cases were clustered, with 70% occurring in 18% of households.

Herrer, in the only other published epidemiological study done in 1953 [47], measured serial culture positivity in a group of students who had recently come to live in an endemic area. He found that 29% (4 of 14) had asymptomatic infection but that 2 failed to produce positive cultures on repeated testing. He concluded that this transient bacteraemia makes it less likely that asymptomatic carriers are a reservoir for infection.

#### The effect of ‘El Niño’

Two studies have looked specifically at the effect of the ‘El Niño’ phenomenon on the number of cases of bartonellosis. One study [48] compared numbers of cases between pre-, post- and 1997–1998 ‘El Niño’ periods in the Cusco and Ancash areas and found that sea surface temperature correlated significantly with the number of cases of Carrion's disease.

The other study, by Chinga-Alayo et al. [13] described similar findings during the 1983–1988 event in Ancash with an almost 4-fold increase in monthly cases.

#### Reservoirs

Due to the sporadic nature of Carrion's disease outbreaks, various candidate reservoirs have been postulated in a number of studies. Herrer [49], in 1953, proposed that euphorb plants, which are found in great abundance in verruga zones and yield a milky latex, may be a natural reservoir. However, in his study he failed to recover *B. bacilliformis* from the plants, infect seedlings or grow the microorganism in the presence of latex dilutions.

Birtles [50] investigated the possibility of intradomicilliary animals being reservoirs by testing 50 animals from the homes of 11 children who had recently had the illness. Bartonella-like organisms were isolated from 4 out of 9 small non-domesticated rodents trapped inside the houses but they were not confirmed to be *B. bacilliformis*.

Cooper et al. carried out two case-control studies [51], [52] in Zamora Chinchipe province, Ecuador, using questionnaires to determine whether there was an excess of dead or dying animals in case households compared to controls. Case households reported seeing significantly more dead or dying rodents [51] and chickens [52] than controls.

## Discussion

### Diagnosis

The Standards for the Reporting of Diagnostic Accuracy Studies (STARD) checklist [18] states that good quality diagnostic studies must describe the study population, their recruitment (inclusion and exclusion criteria) and sampling method. They must also use a reference standard and describe the definition of and rationale for the cut-offs of index tests and reference standards used.

Although these standards were published after some of these studies were conducted, it is useful to note that the majority of diagnostic studies were observational studies that lacked many or all of the currently accepted criteria. Sampling methods were not explained in any of the studies, making it difficult to exclude selection bias, and the derivation of cut-offs for tests were not explicitly explained, also potentially introducing bias. In cases where a reference standard was used, this test varied from study to study, making it very difficult to make meaningful comparisons between reports.

Though a positive blood smear, blood culture or histopathological finding can be considered diagnostic of Carrion's disease the lack of any of these does not necessarily exclude the diagnosis. All diagnostic studies are to a degree therefore hampered by the lack of an agreed reference standard and an important initial step in future research efforts should be the agreement of a consensus case definition.

The studies that reported the highest specificity were one looking at ELISA [22] and another at sonicated immunoblot [24]. ELISA had a sensitivity of 85% and a specificity of 100% for detecting both acute and chronic cases. However, the sensitivity of immunoblot for detecting acute cases was only 70% at best and even though the specificity of test was quoted as 100% the test then cross-reacted with a number of other antigens.

IFA proved to have a good sensitivity and specificity, and the study by Chamberlin [19] was classified as 3b, however its use may be more appropriate in detecting past infection, rather than acute diagnosis, as according to Chamberlin's study, 45% of the population in endemic areas may have antibodies without necessarily having symptoms. This is also the case with ELISA and immunoblot where antibodies may remain positive for some time.

The studies on PCR [21] and Western Blot [23], [27] showed 100% detection of acute cases (but 0% of chronic cases in the case of PCR) and 100% specificity, however their sample sizes were very small and lacked the majority of the currently accepted STARD criteria [18].

The other problem with studies looking at these newer high-tech diagnostic techniques is that no information was provided on the cost, implementability and sustainability in endemic regions where facilities are limited and the budget finite.

Blood smears are the cheapest and quickest method of diagnosis for Carrion's disease and are provided free of charge in the Caraz region. However, although their specificity is very high, their sensitivity is very low.

A new diagnostic tool that could also be used for disease surveillance would be a very valuable addition to the armamentarium, though the evaluation of a tool would clearly require a consensus reference case definition to enable performance characteristics to be defined.

### Management

The literature on the management of acute and chronic Carrion's disease consists mainly of in vitro studies or case series. This implies that our current treatment guidelines are supported by level 4 and 5 evidence. In addition, the majority of these studies lack a definition of cure which is problematic in Carrion's disease as although patients may be clinically ‘cured,’ they may still have organisms in the circulation and therefore potentially be contributing to the transmission of the disease. Instead, the majority of the studies used the proportion of patients who made a ‘good recovery’ to define whether an antibiotic was effective or not. Not only is this an unsatisfactory way of determining treatment effectiveness but also, authors did not define what they meant by ‘good recovery.’ These issues make it difficult to draw meaningful conclusions from these studies.

#### Acute disease

Only studies looking at quinolones and chloramphenicol were found in the literature search but none comparing the two.

There has been much discussion recently about the use of quinolones in the management of acute Carrion's disease due to the publication of a number of experimental studies suggesting constitutive expression of gyrA mutations which confer quinolone resistance. However, in vivo studies looking at this class of antimicrobials are lacking and therefore it is difficult to draw meaningful conclusions from these findings.

Although these are in vitro studies, and therefore evidence level 5, some go on to claim that ciprofloxacin is inadequate [28], [30], [32] and should be removed from the current guidelines. One of these studies [32] goes on to give an example of an in vivo case of ciprofloxacin failure who died, however this patient was extremely sick on presentation and did not recover despite simultaneously receiving ciprofloxacin and ceftriaxone iv [53]. There is also some discrepancy between studies in acceptable MICs for antibiotics with some claiming that 0.25 makes ciprofloxacin inadequate [30] and others claiming that a MIC of 0.25 makes it highly effective [29].

The evidence behind the use of chloramphenicol is only slightly more established, with two level 4 observational studies [34], [35] claiming a 79% and 89% recovery rate respectively and a minireview [33] indicating that there is good evidence for its use based on two small observational studies.

#### Chronic disease

The unpublished results of the study carried out by a team of Caraz researchers led by Jesus Gonzalez [37] are very compelling as this is the first study to compare two antibiotics and the only study to use microbiological cure as the end-point. Its results, which show that a weekly 1 g azithromycin dose is just as effective as daily rifampicin, has important implications. Not only could the weekly dose improve adherence but it would avoid the indiscriminant use of an anti-tuberculous drug in an area where multi-drug resistant (MDR) TB is a problem.

Rifampicin is now the most widely used antibiotic in the chronic phase and most patients have been found to respond well; 80% and 93.1% respectively in two studies one of which was a minireview and therefore level 3a evidence [23], [35].

Studies looking at less-conventional treatments; large blood transfusions for acute disease [36], and sultamicillin and deflazacort for verruga peruana [38], involved only two and one patient respectively and therefore their use in practice cannot be determined.

### Vectors and control

Though species of Lutzomyia are believed to be the principal vector this assumption belies a marked lack of evidence.

*B. bacilliformis* was isolated from *L. peruensis* in the Cusco area [25]. However the incrimination of *L. robusta* and *L. maranonensis* as vectors in Jaen, San Ignacio and Utcubamba cannot yet be confirmed. Similarly, *L. serrano* may be responsible for transmitting Carrion's disease in the high jungle area of Peru [41].

*L. verrucarum* has long been the presumed vector in the highly endemic areas of Caraz and its surroundings. Although experiments carried out to date have not yet established whether trans-ovarial transmission of *B. bacilliformis* occurs in this species, it seems that highly bacteraemic blood is detrimental to the sandfly, though the significance of this is yet to be determined [42].

The studies on control are of very low evidence categories but show that both DDT and pyrethroids are effective in reducing sandfly populations in endemic areas. The study in Caraz [44] additionally provides possible hypotheses for their continued efficacy past the insecticide lifespan. Research looking at these potential additional properties has not yet been carried out but the idea is compelling and could allow a more cost-effective control strategy to be designed for use in endemic areas

### Epidemiology and prevention

The epidemiological studies done to date have provided us with crude figures for incubation period (20 to 100 days) [45], experimented with primitive vaccines which showed some possible protection [46], and determined that significantly more cases of Carrion's disease occur during ‘El Niño’ periods [14], [48].

However, one study above all has provided crucial epidemiological figures for Carrion's disease in the highly endemic area of Caraz. The study by Chamberlin et al. [13] which had rigorous methodology and therefore classified as level 1b evidence, provided the first accurate figures for incidence and prevalence of past infection, though the point prevalence of asymptomatic bacteraemia of 0.5% was lower than previously thought, casting doubt on the theory that humans are the sole reservoir.

Studies looking at alternative reservoirs are mostly inconclusive, with euphorb plants [48] and intra-domicilliary animals [49]–[51] failing to contain *B. bacilliformis* in a number of experiments. Cooper's studies in Ecuador, which found that households with cases reported more dead or dying rodents and chickens, are also unreliable (evidence level 4) as none of the animals were tested for *B. bacilliformis* and the questionnaire methodology is potentially subject to bias including responder bias if questions were asked in a leading way by the interviewers who were not blind to whether households were case or control.

### Implications for practice

This work has provided an insight into what is known about Carrion's disease by looking at the highest-level evidence, and has highlighted the important knowledge gaps which need to be addressed if control efforts are to have any chance of success.

Diagnostic methods remain unsatisfactory in this disease, with those that are cheap and readily available having a low sensitivity and those that have a potentially higher sensitivity being more expensive and impractical in most health centres in endemic regions. New tools are needed and future evaluations require rigour and clear case and outcome definitions. Optimal case and outbreak management remains entirely unclear at present though azithromycin may emerge as an excellent option.

### Implications for research

Most of the studies included in the systematic review are level 4 or 5, indicating that there is a lack of high-level evidence guiding our current practice in Carrion's disease.

Development of effective surveillance tools to inform understanding of the epidemiology of disease (both clinical and subclinical), harmonized case and outcome definitions, readiness for outbreak investigation including evaluation of (putative) vector control strategies, contact investigations and environmental and reservoir host studies will all be important. Evaluation of treatment strategies for individuals, households and perhaps communities might also be worth pursuing. Understanding of immunity and strain diversity is vestigial and advances might yield important insights into pathophysiology.

There may be an opportunity to eliminate this ancient disease which has been the scourge of poor, rural, mountain communities for centuries but the expanding ecology of the putative vector may limit the time available to seize this opportunity. An intensive multidisciplinary research effort could yield the tools and strategies required for success and the demonstration of this approach could serve as a blueprint for other geographically-bound infectious diseases. Daniel Carrion would be turning in his mausoleum beneath Dos de Mayo Hospital in Lima if he only knew how little we have progressed since his first (and last) big advance.

### Study limitations

It was not possible to access some historical journals and there were a number of articles published in smaller journals that were not able to be accessed due to time and financial constraints. This has unavoidably excluded some articles from the systematic review.

## Supporting Information

Figure S1 **PRISMA flow diagram.**
(DOC)

Figure S2 **PRISMA checklist.**
(DOC)

Figure S3 **Oxford Centre for Evidence-based Medicine – Levels of Evidence.**
(DOCX)
