# Supplementary material for: Bartonella bacilliformis: A Systematic Review of the Literature to Guide the Research Agenda for Elimination
Source: PLoS Negl Trop Dis. 2012 Oct 25;6(10):e1819. doi: 10.1371/journal.pntd.0001819 (PMC3493376; doi:10.1371/journal.pntd.0001819)
Supplement: Figure S1 — PRISMA flow diagram. (DOC) [file pntd.0001819.s001.doc]

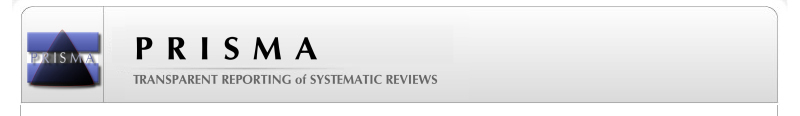
**PRISMA 2009 Flow Diagram**

**Screening**

**Included**

**Eligibility**

**Identification**

Records identified through database searching
(n = 428 )

Additional records identified through other sources
(n =58 )

Records after duplicates removed
(n = 480 )

Records screened
(n = 480 )

Records excluded
(n = 356 )

Full-text articles assessed for eligibility
(n =124)

9)

Full-text articles excluded, with reasons
(n = 77 )

Studies included in qualitative synthesis
(n = 47 )

Studies included in quantitative synthesis (meta-analysis)
(n = 0 )
