# Supplementary material for: Bartonella bacilliformis: A Systematic Review of the Literature to Guide the Research Agenda for Elimination
Source: PLoS Negl Trop Dis. 2012 Oct 25;6(10):e1819. doi: 10.1371/journal.pntd.0001819 (PMC3493376; doi:10.1371/journal.pntd.0001819)
Supplement: Figure S3 — Oxford Centre for Evidence-based Medicine – Levels of Evidence. (DOCX) [file pntd.0001819.s003.docx]

Oxford Centre for Evidence-based Medicine - Levels of Evidence

| **Level** | **Therapy / Prevention, Aetiology / Harm** | **Prognosis** | **Diagnosis** | **Differential diagnosis / symptom prevalence study** | **Economic and decision analyses** |
| --- | --- | --- | --- | --- | --- |
| 1a | SR (with homogeneity*) of RCTs | SR (with homogeneity*) of inception cohort studies; CDR" validated in different populations | SR (with homogeneity*) of Level 1 diagnostic studies; CDR" with 1b studies from different clinical centres | SR (with homogeneity*) of prospective cohort studies | SR (with homogeneity*) of Level 1 economic studies |
| 1b | Individual RCT (with narrow Confidence Interval"¡) | Individual inception cohort study with > 80% follow-up; CDR" validated in a single population | Validating** cohort study with good" " " reference standards; or CDR" tested within one clinical centre | Prospective cohort study with good follow-up**** | Analysis based on clinically sensible costs or alternatives; systematic review(s) of the evidence; and including multi-way sensitivity analyses |
| 1c | All or none§ | All or none case-series | Absolute SpPins and SnNouts" " | All or none case-series | Absolute better-value or worse-value analyses " " " " |
| 2a | SR (with homogeneity*) of cohort studies | SR (with homogeneity*) of either retrospective cohort studies or untreated control groups in RCTs | SR (with homogeneity*) of Level >2 diagnostic studies | SR (with homogeneity*) of 2b and better studies | SR (with homogeneity*) of Level >2 economic studies |
| 2b | Individual cohort study (including low quality RCT; e.g., <80% follow-up) | Retrospective cohort study or follow-up of untreated control patients in an RCT; Derivation of CDR" or validated on split-sample§§§ only | Exploratory** cohort study with good" " " reference standards; CDR" after derivation, or validated only on split-sample§§§ or databases | Retrospective cohort study, or poor follow-up | Analysis based on clinically sensible costs or alternatives; limited review(s) of the evidence, or single studies; and including multi-way sensitivity analyses |
| 2c | "Outcomes" Research; Ecological studies | "Outcomes" Research |  | Ecological studies | Audit or outcomes research |
| 3a | SR (with homogeneity*) of case-control studies |  | SR (with homogeneity*) of 3b and better studies | SR (with homogeneity*) of 3b and better studies | SR (with homogeneity*) of 3b and better studies |
| 3b | Individual Case-Control Study |  | Non-consecutive study; or without consistently applied reference standards | Non-consecutive cohort study, or very limited population | Analysis based on limited alternatives or costs, poor quality estimates of data, but including sensitivity analyses incorporating clinically sensible variations. |
| 4 | Case-series (and poor quality cohort and case-control studies§§) | Case-series (and poor quality prognostic cohort studies***) | Case-control study, poor or non-independent reference standard | Case-series or superseded reference standards | Analysis with no sensitivity analysis |
| 5 | Expert opinion without explicit critical appraisal, or based on physiology, bench research or "first principles" | Expert opinion without explicit critical appraisal, or based on physiology, bench research or "first principles" | Expert opinion without explicit critical appraisal, or based on physiology, bench research or "first principles" | Expert opinion without explicit critical appraisal, or based on physiology, bench research or "first principles" | Expert opinion without explicit critical appraisal, or based on economic theory or "first principles" |

| * | By homogeneity we mean a systematic review that is free of worrisome variations (heterogeneity) in the directions and degrees of results between individual studies. Not all systematic reviews with statistically significant heterogeneity need be worrisome, and not all worrisome heterogeneity need be statistically significant. As noted above, studies displaying worrisome heterogeneity should be tagged with a "-" at the end of their designated level. |
| --- | --- |
| " | Clinical Decision Rule. (These are algorithms or scoring systems that lead to a prognostic estimation or a diagnostic category.) |
| "¡ | See note above for advice on how to understand, rate and use trials or other studies with wide confidence intervals. |
| § | Met when all patients died before the Rx became available, but some now survive on it; or when some patients died before the Rx became available, but none now die on it. |
| §§ | By poor quality cohort study we mean one that failed to clearly define comparison groups and/or failed to measure exposures and outcomes in the same (preferably blinded), objective way in both exposed and non-exposed individuals and/or failed to identify or appropriately control known confounders and/or failed to carry out a sufficiently long and complete follow-up of patients. By poor quality case-control study we mean one that failed to clearly define comparison groups and/or failed to measure exposures and outcomes in the same (preferably blinded), objective way in both cases and controls and/or failed to identify or appropriately control known confounders. |
| §§§ | Split-sample validation is achieved by collecting all the information in a single tranche, then artificially dividing this into "derivation" and "validation" samples. |
| " " | An "Absolute SpPin" is a diagnostic finding whose Specificity is so high that a Positive result rules-in the diagnosis. An "Absolute SnNout" is a diagnostic finding whose Sensitivity is so high that a Negative result rules-out the diagnosis. |
| "¡"¡ | Good, better, bad and worse refer to the comparisons between treatments in terms of their clinical risks and benefits. |
| " " " | Good reference standards are independent of the test, and applied blindly or objectively to applied to all patients. Poor reference standards are haphazardly applied, but still independent of the test. Use of a non-independent reference standard (where the 'test' is included in the 'reference', or where the 'testing' affects the 'reference') implies a level 4 study. |
| " " " " | Better-value treatments are clearly as good but cheaper, or better at the same or reduced cost. Worse-value treatments are as good and more expensive, or worse and the equally or more expensive. |
| ** | Validating studies test the quality of a specific diagnostic test, based on prior evidence. An exploratory study collects information and trawls the data (e.g. using a regression analysis) to find which factors are 'significant'. |
| *** | By poor quality prognostic cohort study we mean one in which sampling was biased in favour of patients who already had the target outcome, or the measurement of outcomes was accomplished in <80% of study patients, or outcomes were determined in an unblinded, non-objective way, or there was no correction for confounding factors. |
| **** | Good follow-up in a differential diagnosis study is >80%, with adequate time for alternative diagnoses to emerge (for example 1-6 months acute, 1 - 5 years chronic) |

Grades of Recommendation:

| A | consistent level 1 studies |
| --- | --- |
| B | consistent level 2 or 3 studies ***or*** extrapolations from level 1 studies |
| C | level 4 studies ***or*** extrapolations from level 2 or 3 studies |
| D | level 5 evidence ***or*** troublingly inconsistent or inconclusive studies of any level |
